# Supplementary material for: Development and validation of a predictive model for postpartum endometritis
Source: PLoS One. 2024 Jul 23;19(7):e0307542. doi: 10.1371/journal.pone.0307542 (PMC11265687; doi:10.1371/journal.pone.0307542)
Supplement: S1 File — (DOCX) [file pone.0307542.s002.docx]

Univariate multivariate logistic regression:

setwd(dir="C:/Users/Administrator/Desktop")

library(readr)

data <- read.csv("train.csv")

names(data)

x1 <- c("Age","BMI","Gestation period","Number of pregnancies","Length of prenatal stay","Prenatal haemoglobin","Prenatal albumin","No. of vaginal finger tests","Duration of membrane breaking","Postpartum haemorrhage")

x2 <- c("Educational attainment","Hypertension in pregnancy","Diabetes in pregnancy","Pre-eclampsia","Maternity methods","Prenatal reproductive tract culture","Mode of rupture of membranes","Balloon induction of labour","Uterine exploration")

library(tableone)

table1 <- CreateTableOne(vars = c(x1,x2),data=data,factorVars = x2,strata = "Statu",addOverall = TRUE)

results1 <- print(table1,showAllLevels = FALSE)

model <- glm(Statu~ BMI + Number of pregnancies + Length of prenatal stay + No. of vaginal finger tests + Duration of membrane breaking + Postpartum haemorrhage + Pre-eclampsia + Maternity methods + Prenatal reproductive tract culture + Uterine exploration,data=data,family=binomial())

summary(model)$coefficients

exp(cbind("OR"=coef(model),confint(model)))

library(foreign)

library(rms)

dd <- datadist(data)

options(datadist="dd")

formula1<-as.formula(Statu~ No. of vaginal finger tests + Postpartum haemorrhage + Pre-eclampsia + Maternity methods + Prenatal reproductive tract culture + Uterine exploration)

fit1<-lrm(formula1,data = data,x=T,y=T)

summary(fit1)

nom1<-nomogram(fit1,fun=function(x)1/(1+exp(-x)),lp=F,fun.at = c(0.1,0.3,0.5,0.7,0.9),funlabel = "Risk")

plot(nom1)

library(regplot)

nom1 <- regplot(fit1,observation=data[3,],center=TRUE,title="Nomogram",points=TRUE,odds=FALSE,showP=TRUE,rank="sd",clickable=FALSE,subticks =TRUE )

Nomogram + the following code = calculate the total score for each patient:

library(nomogramFormula)

results<-formula_rd(nomogram=nom1)

points1 <- points_cal(formula = results$formula, rd = data)

length(points1)

head(points1)

points1

data$points<-points_cal(formula = results$formula,rd=data)

write.csv(data,file = "datapoint.csv")

ROC curve code:

setwd(dir="C:/Users/Administrator/Desktop")

library(readr)

data <- read.csv("train.csv")

library(foreign)

library(rms)

dd <- datadist(data)

options(datadist="dd")

formula1<-as.formula(Statu~ No. of vaginal finger tests + Postpartum haemorrhage + Pre-eclampsia + Maternity methods + Prenatal reproductive tract culture + Uterine exploration)

fit1<-lrm(formula1,data = data,x=T,y=T)

data$predvalue1 <- predict(fit1)

library(pROC)

ROC1 <- roc(data$Statu,data$predvalue1)

round(auc(ROC1),3)

round(ci(auc(ROC1)),3)

plot(1-ROC1$specificities,ROC1$sensitivities,type="l",col="green",lty=1,xlab = "1-specificity",ylab = "sensitivity",lwd=2)

abline(0,1)

legend(0.55,0.35,c("train"),lty = c(1),lwd=c(2),col=c("green"),bty="o")

plot(ROC1, print.auc=TRUE, auc.polygon=TRUE, grid=c(0.1, 0.2),

grid.col=c("green", "red"), max.auc.polygon=TRUE,

auc.polygon.col="lightblue")

Baseline table code:

setwd(dir="C:/Users/Administrator/Desktop")

library(readr)

data <- read.csv("hebi.csv")

names(data)

library(compareGroups)

library(tableone)

data$ Educational attainment <-factor( data$ Educational attainment,levels = c(1, 2),labels = c("yes", "no") )

data$ Hypertension in pregnancy <-factor( data$ Hypertension in pregnancy,levels = c(1, 0),labels = c("yes", "no") )

data$ Diabetes in pregnancy <-factor( data$ Diabetes in pregnancy,levels = c(1, 0),labels = c("yes", "no") )

data$ Pre-eclampsia <-factor( data$ Pre-eclampsia,levels = c(1, 0),labels = c("yes", "no") )

data$ Maternity methods <-factor( data$ Maternity methods,levels = c(1, 2),labels = c("yes", "no") )

data$ Prenatal reproductive tract culture <-factor( data$ Prenatal reproductive tract culture,levels = c(1, 0),labels = c("yes", "no") )

data$ Mode of rupture of membranes <-factor( data$ Mode of rupture of membranes,levels = c(1, 2),labels = c("yes", "no") )

data$ Balloon induction of labour <-factor( data$ Balloon induction of labour,levels = c(1, 0),labels = c("yes", "no") )

data$ Uterine exploration <-factor( data$ Uterine exploration,levels = c(1, 0),labels = c("yes", "no") )

data$Statu <-factor( data$Statu,levels = c(1, 0),labels = c("yes", "no") )

tab1 <- descrTable(group ~.,data = data)

print(tab1)

export2word(tab1,file="table1.docx")

Calibration curve code:

setwd(dir="C:/Users/Administrator/Desktop")

library(readr)

data <- read.csv("train.csv")

library(foreign)

library(rms)

bc <- na.omit(data)

dd <- datadist(data)

options(datadist="dd")

formula1<-as.formula(Statu~ No. of vaginal finger tests + Postpartum haemorrhage + Pre-eclampsia + Maternity methods + Prenatal reproductive tract culture + Uterine exploration)

fit1<-lrm(formula1,data = data,x=T,y=T)

cal1<-calibrate(fit1,method = "boot",B=500)

plot(cal1,xlim = c(0,1),xlab = "Predicted Probability",ylab = "Observed Probability",legend = FALSE,subtitles = FALSE)

abline(0,1,col="black",lty=2,lwd=2)

lines(cal1[,c("predy","calibrated.orig")],type = "l",lwd=2,col="red",pch=16)

lines(cal1[,c("predy","calibrated.corrected")],type = "l",lwd=2,col="green",pch=16)

legend(0.55,0.35,c("Apparent","Ideal","Bias-corrected"),lty=c(2,1,1),lwd=c(2,1,1),col=c("black","red","green"),bty="n")

Decision curve code:

library(rms)

bc <- na.omit(data)

dd <- datadist(data)

options(datadist="dd")

formula1<-as.formula(Statu~ No. of vaginal finger tests + Postpartum haemorrhage + Pre-eclampsia + Maternity methods + Prenatal reproductive tract culture + Uterine exploration)

fit1<-lrm(formula1,data = data,x=T,y=T)

library(rmda)

model_1 <- decision_curve(formula1,data=data,family = binomial(link="logit"),thresholds = seq(0,1,by=0.01),confidence.intervals = 0.95,study.design = "case-control",population.prevalence = 0.3)

plot_decision_curve(model_1,curve.names = c("model_1"),xlim = c(0,1.0),cost.benefit.axis = FALSE,col=c("red"),confidence.intervals =FALSE,standardize = FALSE )

Regression OR value forest plot codes:

setwd(dir="C:/Users/Administrator/Desktop")

library(forestmodel)

library(readr)

data <- read.csv("datapoint.csv")

head(data)

logreg<-glm(Statu~points,family = binomial,data = data)

forest_model(logreg,format_options = forest_model_format_options(colour = "steelblue",shape = 15, text_size = 3.8, point_size = 6, banded = TRUE), factor_separate_line = TRUE )

Hosmer Lemeshow Inspection Code:

setwd(dir="C:/Users/Administrator/Desktop")

library(readr)

data <- read.csv("train.csv")

library(foreign)

library(rms)

dd <- datadist(data)

options(datadist="dd")

formula1<-as.formula(Statu~ No. of vaginal finger tests + Postpartum haemorrhage + Pre-eclampsia + Maternity methods + Prenatal reproductive tract culture + Uterine exploration)

fit1<-glm(formula1,data = data,family=binomial(link=logit))

library(ResourceSelection)

hl1 <- hoslem.test(fit1$y,fitted(fit1),g=10)

hl1

Second method of plotting calibration curves (with brier scores):

setwd(dir="C:/Users/Administrator/Desktop")

library(readr)

data <- read.csv("train.csv")

library(foreign)

library(rms)

bc <- na.omit(data)

dd <- datadist(data)

options(datadist="dd")

formula1<-as.formula(Statu~ No. of vaginal finger tests + Postpartum haemorrhage + Pre-eclampsia + Maternity methods + Prenatal reproductive tract culture + Uterine exploration)

library(riskRegression)

fit1=glm(formula1,data=data,family=binomial())

xb <- Score(list("fit"=fit1),formula=Statu~1,null.model=FALSE,conf.int=TRUE,plots=c("calibration","ROC"),metrics=c("auc","brier"),B=1000,M=50,data=data)

plotCalibration(xb,col="red")

Random Forest Code:

library(readr)

setwd(dir="C:/Users/Administrator/Desktop")

data <- read.csv("train.csv")

data <- na.omit(data)

data$Statu <- factor(data$Statu,levels = c(0,1),labels = c("Non- Endometritis "," Endometritis "))

data$ Maternity methods <- factor(data$ Maternity methods,levels = c(1,2),labels = c("Natural birth "," Cesarean section "))

data$ Educational attainment <- factor(data$ Educational attainment,levels = c(1,2),labels = c("Junior high below "," Junior high above "))

data$ Hypertension in pregnancy <- factor(data$ Hypertension in pregnancy,levels = c(0,1),labels = c("No "," Yes "))

data$ Diabetes in pregnancy <- factor(data$ Diabetes in pregnancy,levels = c(0,1),labels = c("No "," Yes "))

data$ Prenatal reproductive tract culture <- factor(data$ Prenatal reproductive tract culture,levels = c(0,1),labels = c("Germ-free "," Germ-carrying "))

data$ Mode of rupture of membranes <- factor(data$ Mode of rupture of membranes,levels = c(1,2),labels = c("Not man-made "," Man-made "))

data$ Balloon induction of labour <- factor(data$ Balloon induction of labour,levels = c(0,1),labels = c("No "," Yes "))

data$ Uterine exploration <- factor(data$ Uterine exploration,levels = c(0,1),labels = c("No "," Yes "))

data$ Pre-eclampsia <- factor(data$ Pre-eclampsia,levels = c(0,1),labels = c("No "," Yes "))

View(data)

summary(data)

str(data)

install.packages("randomForest")

library(randomForest)

set.seed(123)

model <- randomForest(Statu~.,data=data)

model

plot(model)

which.min(model$err.rate[,1])

model2 <- randomForest(Statu~.,data=data,ntree=384,mtry=4)

print(model2)

importance(model2)

varImpPlot(model2)

library(ggplot2)

library(RColorBrewer)

library(tidyverse)

library(randomForest)

library(rfPermute)

library(openxlsx)

setwd(dir="C:/Users/Administrator/Desktop")

library(readr)

df <- read.csv("train1.csv")

set.seed(123)

df.rf <- randomForest(Statu~.,data=df,ntree=1000,importance=TRUE,proximity=TRUE)

importance(df.rf,decreasing=F)

set.seed(123)

Statu.rfP <- rfPermute(Statu~.,data=df,ntree=1000,nrep=299,num.cores = 3)

importance(Statu.rfP,decreasing=T)

Statu.data <- data.frame(importance(Statu.rfP,decreasing = T))

view(Statu.data)

Statu.data <- mutate(Statu.data,label=ifelse(X.IncMSE.pval < 0.001,'***',ifelse(X.IncMSE.pval < 0.01,'**',ifelse(X.IncMSE.pval < 0.05,'*',''))))

view(Statu.data)

Statu.data$name <- rownames(Statu.data)

View(Statu.data)

Statu.data$name <- factor(Statu.data$name,levels =Statu.data$name)

View(Statu.data)

P <- ggplot(Statu.data,aes(name,X.IncMSE))+geom_bar(aes(fill=label),stat ='identity')+scale_fill_manual(values = brewer.pal(6,"Accent"))+geom_text(aes(y=X.IncMSE+0.5,label=label))+theme_classic()+labs(x='',y='Increase in MSE(%)')+theme(legend.position='')+coord_flip()

P

Lasso regression code:

getwd()

setwd(dir="C:/Users/Administrator/Desktop")

getwd()

read.csv("train.csv")

library(readr)

bc <- read_csv("train.csv",locale=locale(encoding="GBK"))

library(glmnet)

library(foreign)

bc <- na.omit(bc)

View(bc)

y<-as.matrix(bc[,21])

x<-as.matrix(bc[,c(2:20)])

f1 = glmnet(x, y, family="binomial", nlambda=100, alpha=1)

print(f1)

plot(f1, xvar="lambda", label=TRUE)

cvfit=cv.glmnet(x,y)

plot(cvfit)

cvfit$lambda.min

cvfit$lambda.1se

l.coef2<-coef(cvfit$glmnet.fit,s=0.009246546,exact = F)

l.coef1<-coef(cvfit$glmnet.fit,s=0.03401231,exact = F)

l.coef1

l.coef2

mod<-glm(Statu~ BMI + Number of pregnancies + Length of prenatal stay + No. of vaginal finger tests + Duration of membrane breaking + Postpartum haemorrhage + Pre-eclampsia + Maternity methods + Prenatal reproductive tract culture + Uterine exploration,family="binomial",data = bc)

summary(mod)

exp(confint(mod))

exp(coef(mod))

Subgroup analysis code:

setwd(dir="C:/Users/Administrator/Desktop")

install.packages('jstable')

library(jstable)

library(readr)

data <- read.csv("train.csv")

View(data)

str(data)

names(data)

library(tidyverse)

df <- data %>%

select(Statu, Educational attainment, Pre-eclampsia, Maternity methods, Uterine exploration) %>%

mutate(Educational attainment =factor(Educational attainment, levels=c(1,2),labels=c("Junior high below "," Junior high above ")),

Pre-eclampsia =factor(Pre-eclampsia, levels=c(0,1),labels=c("No","Yes")),

Maternity methods =factor(Maternity methods, levels=c(1,2),labels=c("Natural birth "," Cesarean section ")),

Uterine exploration =factor(Uterine exploration, levels=c(0,1),labels=c("No","Yes")))

View(df)

str(df)

names(df)

library(jstable)

res <- TableSubgroupMultiGLM(

formula = Statu ~ Maternity methods,

var_subgroups = c("Educational attainment "," Pre-eclampsia "," Uterine exploration "),

data = df )

res

plot_df <- res[,c("Variable","Count","OR","Lower","Upper","P value",

"P for interaction")]

plot_df

plot_df$` ` <- paste(rep("", nrow(plot_df)), collapse = "")

plot_df[,2:7] <- apply(plot_df[,2:7],2,as.numeric)

plot_df[,c(2,6,7)][is.na(plot_df[,c(2,6,7)])] <- ""

install.packages("forestploter")

install.packages("grid")

library(forestploter)

library(grid)

p <- forest(

data = plot_df[,c(1,2,8,6,7)],

lower = plot_df$Lower,

upper = plot_df$Upper,

est = plot_df$OR,

ci_column = 3,

sizes = (plot_df$OR+0.001)*0.3,

ref_line = 1,

xlim = c(0,4))

print(p)

Covariance diagnostic code:

setwd(dir="C:/Users/Administrator/Desktop")

install.packages("car")

library(car)

data <- read.csv("train.csv")

str(data)

model <- lm(Statu~ BMI + Number of pregnancies + Length of prenatal stay + No. of vaginal finger tests + Duration of membrane breaking + Postpartum haemorrhage + Pre-eclampsia + Maternity methods + Prenatal reproductive tract culture + Uterine exploration, data = data)

vif(model)

vif_values <- vif(model)

bar_colors <- ifelse(vif_values >= 2, "red", "green")

plot(vif_values,

col = ifelse(vif_values >= 2, "red", "green"),

ylim = c(0, 5),

pch = ifelse(vif_values >= 2, 15, 16),

cex = 1.5,

main = "VIF Plot",

xlab = "Variables", ylab = "VIF Values")

New subgroup analysis code:

setwd(dir="C:/Users/Administrator/Desktop")

install.packages('jstable')

library(jstable)

library(readr)

data <- read.csv("train.csv")

View(data)

str(data)

names(data)

library(tidyverse)

df <- data %>%

select(Statu, Educational attainment, Pre-eclampsia, Maternity methods, Prenatal reproductive tract culture, No. of vaginal finger tests, Postpartum haemorrhage, Uterine exploration) %>%

mutate(No. of vaginal finger tests =ifelse(No. of vaginal finger tests >5,">5","<=5"),

No. of vaginal finger tests =factor(No. of vaginal finger tests,levels=c(">5","<=5")),

Postpartum haemorrhage =ifelse(Postpartum haemorrhage >350,">350","<=350"),

Postpartum haemorrhage =factor(Postpartum haemorrhage,levels=c(">350","<=350")),

Prenatal reproductive tract culture =factor(Prenatal reproductive tract culture, levels=c(0,1),labels=c("Germ-free "," Germ-carrying ")),

Educational attainment =factor(Educational attainment, levels=c(1,2),labels=c("Junior high below "," Junior high above ")),

Pre-eclampsia =factor(Pre-eclampsia, levels=c(0,1),labels=c("No","Yes")),

Maternity methods =factor(Maternity methods, levels=c(1,2),labels=c("Natural birth "," Cesarean section ")),

Uterine exploration =factor(Uterine exploration, levels=c(0,1),labels=c("No","Yes")))

View(df)

str(df)

names(df)

library(jstable)

res <- TableSubgroupMultiGLM(

formula = Statu ~ Educational attainment,

var_subgroups = c("Maternity methods "," Pre-eclampsia "," Uterine exploration "," Prenatal reproductive tract culture "," No. of vaginal finger tests "," Postpartum haemorrhage "),

data = df )

res

plot_df <- res[,c("Variable","Count","OR","Lower","Upper","P value",

"P for interaction")]

plot_df

plot_df$` ` <- paste(rep("", nrow(plot_df)), collapse = "")

plot_df[,2:7] <- apply(plot_df[,2:7],2,as.numeric)

plot_df[,c(2,6,7)][is.na(plot_df[,c(2,6,7)])] <- ""

install.packages("forestploter")

install.packages("grid")

library(forestploter)

library(grid)

p <- forest(

data = plot_df[,c(1,2,8,6,7)],

lower = plot_df$Lower,

upper = plot_df$Upper,

est = plot_df$OR,

ci_column = 3,

sizes = (plot_df$OR+0.001)*0.3,

ref_line = 1,

xlim = c(0,4))

print(p)

Subgroups by the number of pregnancies:

setwd(dir="C:/Users/Administrator/Desktop")

install.packages('jstable')

library(jstable)

library(readr)

data <- read.csv("vagen.csv")

View(data)

str(data)

names(data)

library(tidyverse)

df <- data %>%

select(Statu, No. of vaginal finger tests, Pre-eclampsia, Maternity methods, Prenatal reproductive tract culture, Number of pregnancies, Postpartum haemorrhage, Uterine exploration) %>%

mutate(Number of pregnancies =ifelse(Number of pregnancies >2,">2","<=2"),

Number of pregnancies =factor(Number of pregnancies,levels=c(">2","<=2")),

No. of vaginal finger tests =ifelse(No. of vaginal finger tests >5,">5","<=5"),

No. of vaginal finger tests =factor(No. of vaginal finger tests,levels=c(">5","<=5")),

Postpartum haemorrhage =ifelse(Postpartum haemorrhage >350,">350","<=350"),

Postpartum haemorrhage =factor(Postpartum haemorrhage,levels=c(">350","<=350")),

Prenatal reproductive tract culture =factor(Prenatal reproductive tract culture, levels=c(1,0),labels=c("Germ-carrying "," Germ-free ")),

Pre-eclampsia =factor(Pre-eclampsia, levels=c(1,0),labels=c("Yes","No")),

Maternity methods =factor(Maternity methods, levels=c(2,1),labels=c("Cesarean section "," Natural birth ")),

Uterine exploration =factor(Uterine exploration, levels=c(1,0),labels=c("Yes","No")))

View(df)

str(df)

names(df)

library(jstable)

res <- TableSubgroupMultiGLM(

formula = Statu ~ Number of pregnancies,

var_subgroups = c("Maternity methods "," Pre-eclampsia "," Uterine exploration "," Prenatal reproductive tract culture "," No. of vaginal finger tests "," Postpartum haemorrhage "),

data = df )

res

plot_df <- res[,c("Variable","Count","OR","Lower","Upper","P value",

"P for interaction")]

plot_df

plot_df$` ` <- paste(rep("", nrow(plot_df)), collapse = "")

plot_df[,2:7] <- apply(plot_df[,2:7],2,as.numeric)

plot_df[,c(2,6,7)][is.na(plot_df[,c(2,6,7)])] <- ""

install.packages("forestploter")

install.packages("grid")

library(forestploter)

library(grid)

p <- forest(

data = plot_df[,c(1,2,8,6,7)],

lower = plot_df$Lower,

upper = plot_df$Upper,

est = plot_df$OR,

ci_column = 3,

sizes = (plot_df$OR+0.001)*0.3,

ref_line = 1,

xlim = c(0,4))

print(p)

Subgroups by age:

setwd(dir="C:/Users/Administrator/Desktop")

install.packages('jstable')

library(jstable)

library(readr)

data <- read.csv("vagen.csv")

View(data)

str(data)

names(data)

library(tidyverse)

df <- data %>%

select(Statu, Age, "Maternity methods "," Pre-eclampsia "," Uterine exploration "," Prenatal reproductive tract culture "," No. of vaginal finger tests "," Postpartum haemorrhage ") %>%

mutate(Age =ifelse(Age >30,">30","<=30"),

Age =factor(Age,levels=c(">30","<=30")),

No. of vaginal finger tests =ifelse(No. of vaginal finger tests >5,">5","<=5"),

No. of vaginal finger tests =factor(No. of vaginal finger tests,levels=c(">5","<=5")),

Postpartum haemorrhage =ifelse(Postpartum haemorrhage >350,">350","<=350"),

Postpartum haemorrhage =factor(Postpartum haemorrhage,levels=c(">350","<=350")),

Prenatal reproductive tract culture =factor(Prenatal reproductive tract culture, levels=c(1,0),labels=c("Germ-carrying "," Germ-free ")),

Pre-eclampsia =factor(Pre-eclampsia, levels=c(1,0),labels=c("Yes","No")),

Maternity methods =factor(Maternity methods, levels=c(2,1),labels=c("Cesarean section "," Natural birth ")),

Uterine exploration =factor(Uterine exploration, levels=c(1,0),labels=c("Yes","No")))

View(df)

str(df)

names(df)

library(jstable)

res <- TableSubgroupMultiGLM(

formula = Statu ~ Age,

var_subgroups = c("Maternity methods "," Pre-eclampsia "," Uterine exploration "," Prenatal reproductive tract culture "," No. of vaginal finger tests "," Postpartum haemorrhage "), data = df)

res

plot_df <- res[,c("Variable","Count","OR","Lower","Upper","P value",

"P for interaction")]

plot_df

plot_df$` ` <- paste(rep("", nrow(plot_df)), collapse = "")

plot_df[,2:7] <- apply(plot_df[,2:7],2,as.numeric)

plot_df[,c(2,6,7)][is.na(plot_df[,c(2,6,7)])] <- ""

install.packages("forestploter")

install.packages("grid")

library(forestploter)

library(grid)

p <- forest(

data = plot_df[,c(1,2,8,6,7)],

lower = plot_df$Lower,

upper = plot_df$Upper,

est = plot_df$OR,

ci_column = 3,

sizes = (plot_df$OR+0.001)*0.3,

ref_line = 1,

xlim = c(0,4))

print(p)
