# Supplementary material for: Development and validation of a predictive model for postpartum endometritis
Source: PLoS One. 2024 Jul 23;19(7):e0307542. doi: 10.1371/journal.pone.0307542 (PMC11265687; doi:10.1371/journal.pone.0307542)
Supplement: Attachment — Submitted filename: Response to Reviewers.docx [file pone.0307542.s003.docx]

**Response Letter**

Dear editors and reviewers,

We deeply appreciate your valuable suggestions and comments, which enable us to further improve our paper. We have studied the comments carefully and have made our best efforts to revise the entire paper as suggested by the editor and reviewers. We are very pleased that our paper can be revised, some **detailed responses** are as follows.

**Detailed responses:**

**To editors:**

1. Please ensure that your manuscript meets PLOS ONE's style requirements, including those for file naming.

Response:Thank you for your constructive comments. We have implemented the necessary changes to align the manuscript with PLOS ONE's style guidelines. Your feedback is greatly valued.

2. We note that your Data Availability Statement is currently as follows: All relevant data are within the manuscript and its Supporting Information files. Please confirm at this time whether or not your submission contains all raw data required to replicate the results of your study. Authors must share the “minimal data set” for their submission. PLOS defines the minimal data set to consist of the data required to replicate all study findings reported in the article, as well as related metadata and method.

Response:Thank you sincerely for your comments. We have uploaded the pertinent supplementary information, including the complete raw dataset and the original code. Your feedback is immensely appreciated.

**Reviewer #1:**

1. The results of the author are confusing. Why is the predictive model for endometritis also used to explore its relationship with coronary heart disease? The average age of the cohort participants is 29 years old. Is it likely for them to develop coronary heart disease?

Response:We extend our gratitude to the reviewer for your invaluable feedback. The primary objective of our study was to devise a predictive tool capable of anticipating the onset of postpartum endometritis, aiming to inform the development of preventive and control measures. In consultation with seasoned obstetricians, we meticulously curated a set of parameters for analysis. These included age, body mass index, education level, gestational week, number of pregnancies, and prenatal conditions such as hypertension, diabetes mellitus, and pre-eclampsia. Furthermore, variables encompassed mode of delivery, duration of antenatal hospitalization, results of admission genital tract cultures, prenatal levels of hemoglobin and albumin, frequency of vaginal examinations pre and post-rupture of membranes, duration and method of membrane rupture, induction of labor using balloon catheters, postpartum hemorrhage severity, and whether uterine exploration was conducted.Our comprehensive analysis, considering a total of 19 variables including postpartum hemorrhage severity and uterine exploration, led us to identify several independent risk factors for postpartum endometritis infection. These factors include inadequate intrauterine cleansing, heightened postpartum hemorrhage, cesarean section delivery, positive genital tract culture upon admission, and increased frequency of vaginal examinations. We wish to clarify that there was no mention or association established with coronary heart disease in our findings. We apologize for any confusion that may have arisen due to ambiguity in our earlier description.Thanks again for your advice,which makes the article more perfect.

2. The results obtained from univariate logistic regression cannot be referred to as "independent risk factors."

Response:We express our gratitude to the reviewer for your insightful feedback. Based on their constructive input, we have revised the designation of independent risk factors to simply "risk factors" in accordance with the univariate logistic regression findings. These adjustments have been visually highlighted with color markings for clarity. We sincerely appreciate your contributions and commitment to enhancing the quality of our work.

3. The basis for including these factors should be clearly defined, including how the factors are measured.

Response:We appreciate your valuable feedback and constructive comments. The factors considered in our study underwent thorough deliberation by a panel of experienced obstetricians to ensure their relevance and reliability. Furthermore, we utilized R-Studio software to generate a graphical representation (Fig 4A) depicting the variance inflation factor (VIF) values. The results illustrated that the VIF values of all 19 factors examined are below 2, indicating minimal covariance among these variables. We are grateful for your guidance and insights throughout this process.

4. Did the author use subgroup analysis to verify the robustness of the model?

Response:We sincerely appreciate your constructive feedback. Following your suggestion, we conducted two subgroup analyses of the prediction model, as illustrated in Fig 10A for subgroups categorized by age and Fig 10B for subgroups categorized by the number of pregnancies. The analysis results indicated that the p-value for interaction among each factor exceeded 0.05. This suggests the absence of any subgroup effects, indicating that each factor can be employed to evaluate the risk level of postpartum acute endometritis occurrence across different age groups and number of pregnancies in pregnant women. These findings further reinforce the reliability and strength of the prediction model. we extend our gratitude for your contributions.

5. The author could adopt more accurate and novel methods such as machine learning to construct the predictive model.

Response:We are grateful for your constructive feedback. Following your suggestion, we employed two machine learning methods, namely lasso regression and random forest, to develop the prediction model. Interestingly, the prediction model constructed using lasso regression yielded results identical to those obtained through univariate and multivariate logistic regression (refer to Fig 3 and Table 4). Conversely, while the performance of constructing predictive models using random forests was comparable to that of univariate and multivariate logistic regression (as shown in Fig 2A and Fig 4B), one of the random forest classifications exhibited a notably high error rate (refer to Fig 2B). Consequently, we opted to utilize univariate and multivariate logistic regression for constructing predictive models. We sincerely appreciate your valuable insights and contributions throughout this process.

**Reviewer #2:**

1. Please highlight the main contributions of this work in the Intro section.

Response:We appreciate your constructive feedback. Following your suggestion, we have incorporated the main contributions into the introduction section（Six independent risk factors for postpartum endometritis were ultimately identified through a comprehensive analysis, combining two machine learning approaches with traditional univariate and multifactorial logistic regression analyses. These findings were used to construct nomogram tailored for the swift identification of maternal postpartum patients potentially at high risk of developing postpartum endometritis. Implementing this measure will help mitigate the occurrence of acute postpartum endometritis, consequently alleviating both the physical discomfort and financial strain experienced by pregnant women.）Thank you once again for your valuable comments.

2. Please provide more explanations on the selection of the predictive model.

Response:Thank you for your insightful comments. This study aims to manage the incidence of acute endometritis in postpartum women, with a dichotomous outcome variable influenced by multiple risk factors. Our initial approach involved clinical predictive modeling, with expertise in both univariate and multivariate logistic regression. In response to your feedback, we have enriched the manuscript by incorporating two additional advanced machine learning methods. This not only enhances the validation of our findings but also facilitates our acquisition of advanced knowledge. Once again, we sincerely appreciate your valuable input.

3. Please also clarify comparisons between the predictive model and some other advanced machine learning methods.

Response:We are grateful for your constructive feedback. Following your suggestion, we employed two machine learning methods, namely lasso regression and random forest, to develop the prediction model. Interestingly, the prediction model constructed using lasso regression yielded results identical to those obtained through univariate and multivariate logistic regression (refer to Fig 3 and Table 4). Conversely, while the performance of constructing predictive models using random forests was comparable to that of univariate and multivariate logistic regression (as shown in Fig 2A and Fig 4B), one of the random forest classifications exhibited a notably high error rate (refer to Fig 2B). Consequently, we opted to utilize univariate and multivariate logistic regression for constructing predictive models. We sincerely appreciate your valuable insights and contributions throughout this process.

4. Please present more recent references if they could help to improve the readability of your work, such as: 1. A deep belief network-based clinical decision system for patients with osteosarcoma; and 2. A machine learning-based predictive model for predicting lymph node metastasis in patients with Ewing’s sarcoma.

Response:We appreciate your constructive feedback. Following your suggestions, we have included more recent references in the discussion section. Thank you once again for your valuable comments.

Added references：

1) Li W, Dong Y, Liu W, Tang Z, Sun C, Lowe S, et al.A deep belief network-based clinical decision system for patients with osteosarcoma. Front Immunol. 2022-01-01; 13 1003347. https://doi.org/10.3389/fimmu.2022.1003347 PMID: 36466868
2) Li W, Zhou Q, Liu W, Xu C, Tang ZR, Dong S, et al.A Machine Learning-Based Predictive Model for Predicting Lymph Node Metastasis in Patients With Ewing's Sarcoma. Front Med (Lausanne). 2022-01-01; 9 832108. https://doi.org/10.3389/fmed.2022.832108 PMID: 35463005
3) Li, W, Liu, Y, Liu, W, Tang, ZR, Dong, S, Li, W, et al. Machine Learning-Based Prediction of Lymph Node Metastasis Among Osteosarcoma Patients. Front Oncol. 2022-01-01; 12 797103. https://doi.org/10.3389/fonc.2022.797103 PMID: 35515104

5. Please give more discussions on future works in the final section.

Response:We deeply appreciate your relevant suggestions, which we have incorporated into the discussion section of the manuscript（The high incidence of postpartum acute endometritis (AE) is indeed concerning. Therefore, in the future, we aim to extend the validation of our findings beyond our current scope, encompassing other regions within the province and potentially the entire country. This endeavor will likely entail collaboration with numerous tertiary specialized maternity and child hospitals. Furthermore, we are contemplating the initiation of a prospective cohort study to formulate more comprehensive guidelines for the prevention of postpartum AE.It's worth noting that our study only utilized two machine learning methods. Considering that several studies [21-23] have employed more sophisticated and diverse machine learning techniques to construct and evaluate predictive models, followed by performance comparisons to identify the most effective method, we recognize the value in adopting a similar approach. This could lead to a deeper understanding of the predictive modeling landscape and aid in selecting the optimal machine learning method for model construction.）. You'll find the additions highlighted in red. Once again, thank you for your valuable input.

Although we have made some revisions to the paper, there may be still some problems. We sincerely appreciate your further recommendations to enhance its quality.

Again, we are very grateful for your insightful comments and suggestions, which are extremely valuable and helpful in improving the quality of our manuscript.

Sincerely yours,

Hui Shao

E-mail: 15609631795@163.com
