# Supplementary material for: Development and validation of a predictive model for postpartum endometritis
Source: PLoS One. 2024 Jul 23;19(7):e0307542. doi: 10.1371/journal.pone.0307542 (PMC11265687; doi:10.1371/journal.pone.0307542)
Supplement: Attachment — Submitted filename: Response to Reviewers.docx [file pone.0307542.s004.docx]

**Response Letter**

Dear editors and reviewers,

We deeply appreciate your valuable suggestions and comments, which enable us to further improve our paper. We have studied the comments carefully and have made our best efforts to revise the entire paper as suggested by the editor and reviewers. We are very pleased that our paper can be revised, some **detailed responses** are as follows.

**Detailed responses:**

**To editors:**

1. ACADEMIC EDITOR:

Please respond to all reviewer comments point by point

clarify the added value of your work over the already known

Respond to all issues raised by the statistical review.

Response: Thank you for your valuable feedback. Below, we have addressed each of the reviewers' comments individually. Our study focuses on developing and validating a predictive model for maternal postpartum endometritis using three statistical approaches. The risk assessment for maternal postpartum endometritis infection can be easily implemented clinically through a straightforward column chart. Our research represents a significant advancement in endometritis prevention efforts. We have addressed all statistical review concerns comprehensively. Once again, we appreciate your insightful comments.

2. Additional Editor Comments:

Comments from PLOS Editorial Office: We note that one or more reviewers has recommended that you cite specific previously published works in an earlier round of revision. As always, we recommend that you please review and evaluate the requested works to determine whether they are relevant and should be cited. It is not a requirement to cite these works and you may remove them before the manuscript proceeds to publication. We appreciate your attention to this request.

Response: Thank you for your constructive feedback. We have diligently reviewed and assessed the literature referenced in the revised manuscript. These citations have been included to enhance the quality of our work, and we sincerely appreciate your valuable comments once again.

**To Reviewers:**

**Minor revisions:**
1- Express p-values more precisely than “p< 0.05”.

Response: Thank you for your constructive feedback. We have enhanced the precision of the P-values presented in Tables 3 and 4, clearly marking them in red for clarity(Revisions to lines 147 and 169). Your comments have been greatly appreciated.
2- Statistical analysis section: Typographical error: Fisher’s exact tests.

Response: Thank you for your constructive feedback. We apologize for any oversight. We have rectified the term "Fisher's exact tests" in the statistical analysis section and highlighted the correction in red(Revisions to line 114). Your comments have been instrumental in improving our work, and we appreciate them once again.
3- Model performance evaluation section: Clarify the meaning of “Brier score (0.121 < 0.25) and (0.11 < 0.25)”.

Response: Thank you for your constructive feedback. We have included an explanation of the Brier score (0.121 < 0.25) and (0.11 < 0.25) in the Model Performance Evaluation section, highlighted in red for clarity(Revisions to lines 195-199). Your comments have been greatly appreciated once again.
4- Figure 1: Indicate that the data was randomly split by R software.

Response: Thank you for your constructive feedback. We have illustrated in Figure 1 how the data is randomly split using the sample function in R software(Revisions to line 92). Please refer to the newly uploaded Figure 1 for details. Your comments have been sincerely appreciated once again.
5- Figure 5: Clearly label the probability scale.

Response: Thank you for your constructive feedback. We have enhanced Figure 5 by clearly labeling the probability scales(Revisions to line 205). Please refer to the updated Figure 5 for details. Your comments are greatly appreciated once again.

6- Figure 10: Label the x-axis.

Response: Thank you for your constructive feedback. We have now labeled the X-axis in Figure 10(Revisions to lines 248-250). Please refer to the updated Figure 10 for details. Your comments are much appreciated once again.
7- To assist in the review process, add line numbering to the document.

Response: Thank you for your constructive feedback. We have incorporated line numbering into the document. Your comments are sincerely appreciated once again.

Although we have made some revisions to the paper, there may be still some problems. We sincerely appreciate your further recommendations to enhance its quality.

Again, We appreciate for Editors/Reviewers’ warm work earnestly, and hope the correction will earn your recognition and approval. Once again, thank you very much for your comments and suggestions.

Sincerely yours,

Hui Shao

E-mail: 15609631795@163.com
